# Supplementary material for: Tumorigenicity decrease in Bcl-xL deficient MDCK cells ensuring the safety for influenza vaccine production
Source: PLoS One. 2024 Dec 16;19(12):e0311069. doi: 10.1371/journal.pone.0311069 (PMC11649150; doi:10.1371/journal.pone.0311069)
Supplement: S1 File — (ZIP) [file pone.0311069.s002.zip › S2_File/4_KOG_enrichment/enrichmap/union.DE_gene.subcluster1_KOG_enrichment_map.pdf]

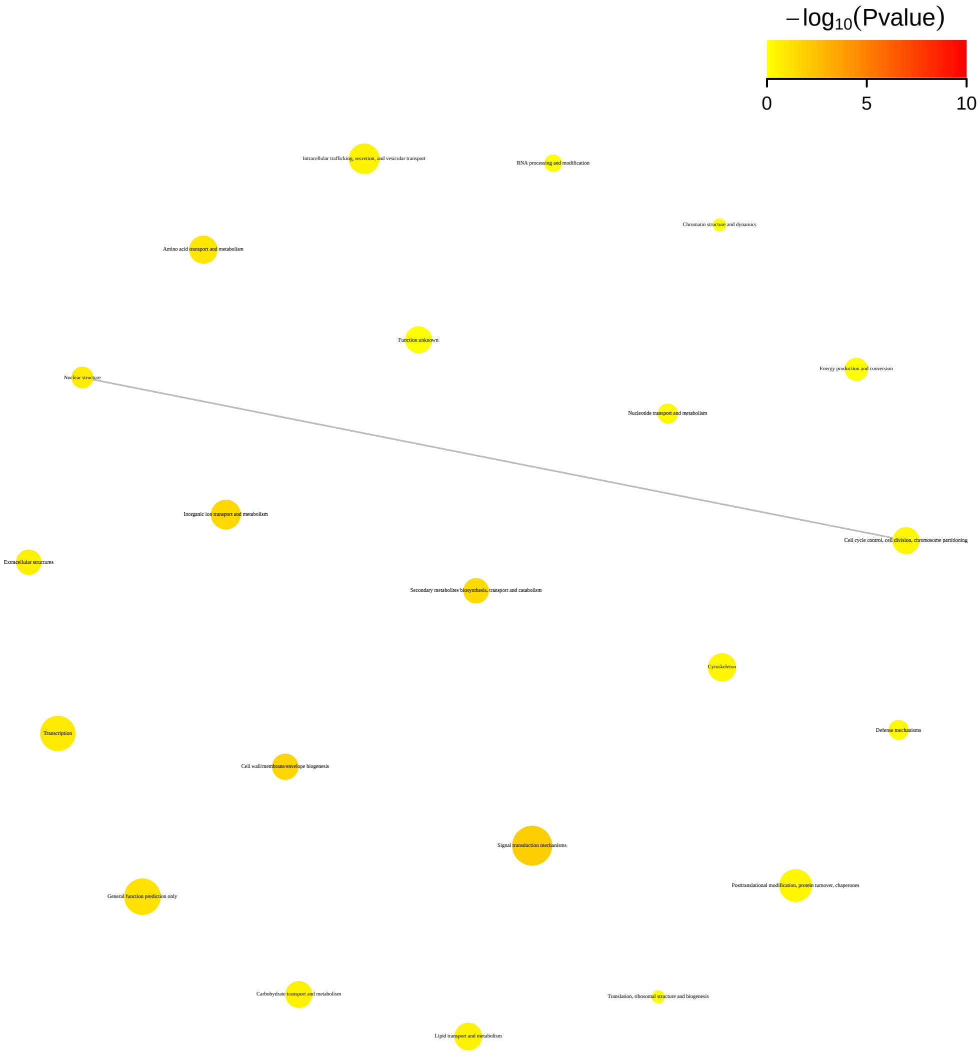

$-\log_{10}(\text{Pvalue})$
0
5
10
Intracellular trafficking, secretion, and vesicular transport
RNA processing and modification
Chromatin structure and dynamics
Amino acid transport and metabolism
Function unknown
Nuclear structure
Energy production and conversion
Nucleotide transport and metabolism
Inorganic ion transport and metabolism
Cell cycle control, cell division, chromosome partitioning
Extracellular structures
Secondary metabolites biosynthesis, transport and catabolism
Cytoskeleton
Transcription
Defense mechanisms
Cell wall/membrane/envelope biogenesis
Signal transduction mechanisms
Posttranslational modification, protein turnover, chaperones
General function prediction only
Carbohydrate transport and metabolism
Translation, ribosomal structure and biogenesis
Lipid transport and metabolism
